# Longitudinal associations of physical activity with plasma metabolites among colorectal cancer survivors up to 2 years after treatment

**DOI:** 10.1038/s41598-021-92279-9

**Published:** 2021-07-02

**Authors:** Eline H. van Roekel, Martijn J. L. Bours, Linda van Delden, Stéphanie O. Breukink, Michèl Aquarius, Eric T. P. Keulen, Audrey Gicquiau, Vivian Viallon, Sabina Rinaldi, Paolo Vineis, Ilja C. W. Arts, Marc J. Gunter, Michael F. Leitzmann, Augustin Scalbert, Matty P. Weijenberg

**Affiliations:** 1 Department of Epidemiology, GROW School for Oncology and Developmental Biology, Maastricht University, P.O. Box 616, 6200 MD Maastricht, The Netherlands; 2 Department of Epidemiology, CAPHRI School for Public Health and Primary Care, Maastricht University, Maastricht, The Netherlands; 3 Department of Surgery, GROW School for Oncology and Developmental Biology & NUTRIM, School of Nutrition and Translational Research in Metabolism, Maastricht University Medical Center+, Maastricht, The Netherlands; 4 Department of Gastroenterology, VieCuri Medical Center, Venlo, the Netherlands; 5 Department of Internal Medicine and Gastroenterology, Zuyderland Medical Centre, Sittard-Geleen, the Netherlands; 6 Biomarkers Group, Nutrition and Metabolism Section, International Agency for Research On Cancer (IARC-WHO), Lyon, France; 7 Nutritional Methodology and Biostatistics Group, Nutrition and Metabolism Section, International Agency for Research On Cancer (IARC-WHO), Lyon, France; 8 MRC Centre for Environment and Health, School of Public Health, Imperial College, London, UK; 9 Italian Institute of Technology, Genoa, Italy; 10 Maastricht Centre for Systems Biology (MaCSBio), Maastricht University, Maastricht, The Netherlands; 11 Department of Epidemiology, CARIM School for Cardiovascular Diseases, Maastricht University, Maastricht, The Netherlands; 12 Nutritional Epidemiology Group, Nutrition and Metabolism Section, International Agency for Research On Cancer (IARC-WHO), Lyon, France; 13 Department of Epidemiology and Preventive Medicine, University of Regensburg, Regensburg, Germany

**Keywords:** Metabolomics, Cancer epidemiology, Gastrointestinal cancer, Epidemiology, Risk factors

## Abstract

We investigated longitudinal associations of moderate-to-vigorous physical activity (MVPA) and light-intensity physical activity (LPA) with plasma concentrations of 138 metabolites after colorectal cancer (CRC) treatment. Self-reported physical activity data and blood samples were obtained at 6 weeks, and 6, 12 and 24 months post-treatment in stage I-III CRC survivors (n = 252). Metabolite concentrations were measured by tandem mass spectrometry (BIOCRATES Absolute*IDQ*p180 kit). Linear mixed models were used to evaluate confounder-adjusted longitudinal associations. Inter-individual (between-participant differences) and intra-individual associations (within-participant changes over time) were assessed as percentage difference in metabolite concentration per 5 h/week of MVPA or LPA. At 6 weeks post-treatment, participants reported a median of 6.5 h/week of MVPA (interquartile range:2.3,13.5) and 7.5 h/week of LPA (2.0,15.8). Inter-individual associations were observed with more MVPA being related (FDR-adjusted q-value < 0.05) to higher concentrations of arginine, citrulline and histidine, eight lysophosphatidylcholines, nine diacylphosphatidylcholines, 13 acyl-alkylphosphatidylcholines, two sphingomyelins, and acylcarnitine C10:1. No intra-individual associations were found. LPA was not associated with any metabolite. More MVPA was associated with higher concentrations of several lipids and three amino acids, which have been linked to anti-inflammatory processes and improved metabolic health. Mechanistic studies are needed to investigate whether these metabolites may affect prognosis.

## Introduction

In 2018, there were almost five million colorectal cancer (CRC) survivors who were diagnosed in the past five years, making CRC the second most prevalent cancer worldwide^1^. The number of CRC survivors is expected to continue to rise in coming years due to a higher incidence and better survival^2^. There is strong evidence that engaging in leisure-time physical activity after a diagnosis of CRC is associated with lower overall^3–5^ and CRC-specific mortality^3,4^, and better quality of life^6–8^. Cancer survivors are currently advised to engage in ≥ 150 min/week of moderate-to-vigorous physical activity (MVPA)^9,10^, including activities expending ≥ 3 metabolic equivalents (METs) such as brisk walking or cycling^11^. Emerging evidence from our research group suggests that light-intensity physical activity (LPA: < 3 METs), e.g. light household work, is also beneficially associated with quality of life among CRC survivors^12^.

The biological mechanisms through which physical activity may influence health and well-being after CRC are not well understood^4,6^. Previous research suggests that obesity, hormones, growth factors, the immune system and inflammatory processes may be involved, but evidence is still preliminary and mostly based on studies in breast cancer survivors^13^. A better understanding of the metabolic pathways that are influenced by physical activity in CRC survivors will contribute to the development of tailored physical activity interventions^14^. Metabolomics is a powerful approach to investigate how health-related exposures and outcomes are associated with human metabolism by allowing a simultaneous assessment of hundreds of metabolites from key metabolic pathways, such as amino acids, phospholipids, sugars and other metabolites^15,16^. To the best of our knowledge, no study to date has applied a metabolomics approach to investigate associations of physical activity with circulating metabolites in CRC survivors. In the general population, cross-sectional associations have been found between physical activity and blood concentrations of phosphatidylcholines (PCs)^17,18^, amino acids^18–24^ and glucose^19,21^. In one of these studies, LPA was associated with several amino acids and glucose, whereas weaker associations were observed for MVPA^19^.

Further research is necessary to determine whether these cross-sectional observations in the general population can be replicated in CRC survivors. Furthermore, a longitudinal analysis is needed to study whether changes in physical activity may affect metabolite concentrations over time, which is important to develop future interventions. Therefore, we investigated longitudinal associations of self-reported time spent in MVPA and LPA with plasma concentrations of 138 targeted metabolites in CRC survivors up to two years post-treatment.

## Methods

### Study design and participants

The Energy for life after ColoRectal cancer (EnCoRe) study is an ongoing prospective cohort study initiated in 2012 (Netherlands Trial Register no. NL6904)^25^. Stage I–III CRC patients are recruited at diagnosis (response ~ 45%) in three participating hospitals in the southeastern part of The Netherlands (Maastricht University Medical Center + , VieCuri Medical Center, and Zuyderland Medical Centre). Men and women aged minimum 18 years old are eligible, while individuals with stage IV CRC and comorbidities obstructing successful study participation (e.g. Alzheimer’s disease) are excluded. Repeated measurements of, among others, self-reported physical activity and blood samples are obtained at diagnosis (before cancer treatment, i.e. surgery, chemotherapy and/or radiotherapy), and at 6 weeks, 6 months, and 1, 2 and 5 years after the end of treatment, by trained research dieticians during home visits. The EnCoRe study has been approved by the Medical Ethics Committee of the Academic Hospital Maastricht and Maastricht University, The Netherlands. The study was conducted in accordance with the Declaration of Helsinki and all participants gave written informed consent.

**Figure 1:**
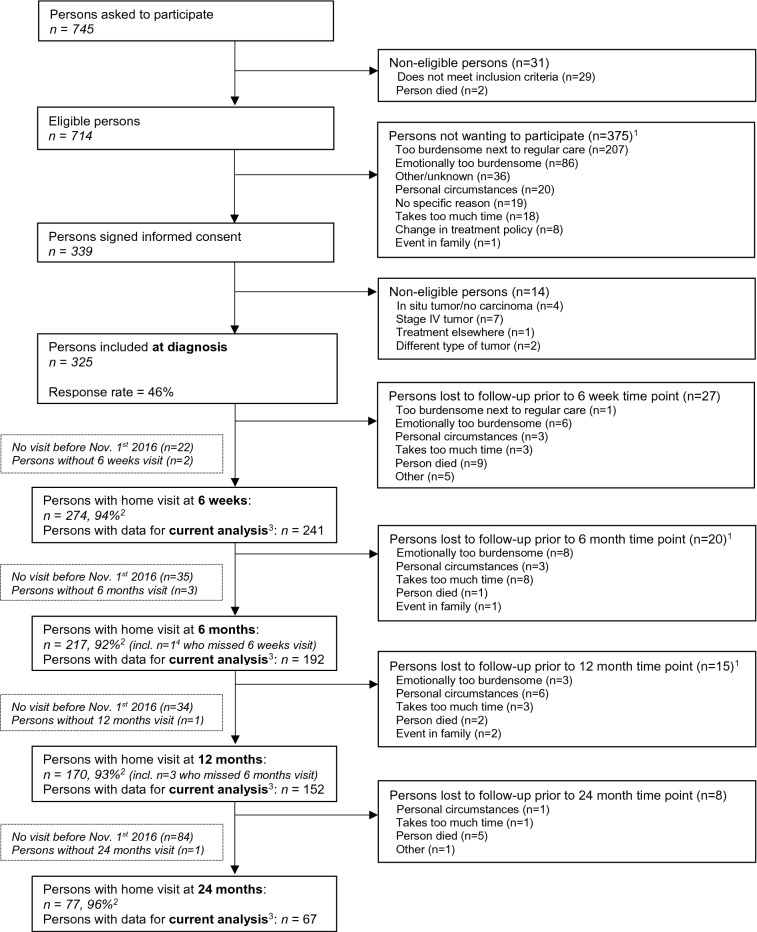
Flow diagram of participants and measurements within the EnCoRe study and the number of post-treatment measurements included in the analyses presented in this paper. Data collected up until November 1st 2016 were included in the analyses. ^1^ Totals do not add up because some individuals reported multiple reasons for non-participation. ^2^ Response rate = (persons with home visits)/(persons with home visits + persons lost to follow-up – persons died). The declining numbers of participants at subsequent time points are predominantly due to the fact that not all participants had reached yet these time points on November 1st 2016. ^3^ Since the current analysis was focused on physical activity and metabolites after colorectal cancer treatment, only post-treatment measurements with available data on self-reported physical activity, metabolites and covariates were included. A total of n = 252 participants with at least one post-treatment measurement including these data were included in the analysis. The number of participants with available physical activity and metabolites data were respectively n = 268 and n = 249 at 6 weeks, n = 215 and n = 203 at 6 months, n = 169 and n = 162 at 1 year, and n = 72 and n = 73 at 2 years post-treatment. ^4^ Other person who also missed 6 weeks visit did not have follow-up visit before November 1st 2016.

For the current study, data collected up until November 1st 2016 were used. Since the study is focused on associations of physical activity and metabolites after CRC treatment, we included individuals with at least one post-treatment measurement of self-reported physical activity, targeted metabolomics and covariates. In total, n = 252 CRC survivors were included in the analyses, with data at 6 weeks (n = 241), 6 months (n = 192), 12 months (n = 152) and 24 months (n = 67) post-treatment (Fig. 1; n = 57 participants had data at all post-treatment time points). The statistical technique being used for longitudinal analysis, i.e. linear mixed modelling, employs all data including data of participants with missing data at some time points^26^. Response rates at post-treatment measurements were > 90% and mortality during follow-up was limited (n = 17). The declining numbers of participants at subsequent time points and the lack of 5 year post-treatment measurements are (predominantly) due to the fact that not all participants included at diagnosis from April 2012 onwards had reached yet these time points in November 2016.

### Physical activity

The Short QUestionnaire to ASsess Health-enhancing physical activity (SQUASH) was used to assess self-reported time spent in MVPA and LPA (hours/week)^27^. Participants reported the frequency (days/week), duration (time/day) and intensity (light, moderate or vigorous) of commuting (walking and cycling), household, work and leisure-time activities (walking, cycling, gardening, odd jobs, and up to four sports) in the previous week. Based on Ainsworth’s Compendium of Physical Activities, all activities were assigned MET-values and categorized as either MVPA (≥ 3 MET; e.g. vigorous household work, walking and sports), or LPA (< 3 MET; e.g. light household or light work activities)^28^. At each time point, the most commonly reported sport was going to the gym. By summing time spent on activities in each category, total hours/week spent in MVPA and LPA were calculated. In addition, it was determined whether participants adhered to the physical activity guidelines (≥ 150 min/week of MVPA)^9,10^. The SQUASH is fairly reliable (test–retest: Spearman’s ρ 0.57–0.58)^27,29^. Its relative validity in assessing time spent in physical activity compared to accelerometer data is comparable to other physical activity questionnaires (Spearman’s ρ 0.56 for total physical activity, 0.35 for vigorous-intensity activities, 0.40 for moderate-intensity activities, and 0.20 for LPA)^29^.

### Blood sampling and laboratory analyses

Blood samples were drawn from fasting participants according to standardized protocols. Samples were collected in 6 mL EDTA plasma tubes, and then centrifuged, aliquoted and stored at − 80 °C within 4 h after blood draw.

Targeted metabolomics analysis were performed at the International Agency for Research on Cancer (IARC), Lyon, France, using the Absolute*IDQ* p180 kit (BIOCRATES Life Sciences AG, Austria) measuring a panel of up to 188 metabolites, including acylcarnitines, amino acids, biogenic amines, a sum of hexoses, PCs including lysoPCs, diacylPCs, and acyl-alkylPCs, and sphingomyelins (SMs). A good to excellent reliability for the majority of metabolites measured using this kit has been observed when comparing samples collected in the same individuals over a period of 4 months^30^ and 2 years^31^ (intra-class correlation coefficients > 0.50 for most metabolites). The procedures and metabolite nomenclature have been described in detail previously^32–34^. Briefly, samples were analyzed by ultra-high performance liquid chromatography (LC; 1290 Series HPLC, Agilent, France) coupled to a tandem mass spectrometer (MS/MS; Triple Quad 4500, AB Sciex, USA). Samples were analyzed in ten analytical batches with participants randomized over batches, and post-treatment samples of each participant placed subsequently in the same batch in a random order. Each batch included human plasma-based quality control (QC) samples from the manufacturer (three spiked samples), from the IARC laboratory (two samples in duplicate), and from a previous cross-sectional study conducted in 2–10 year post-treatment CRC survivors 3 samples in duplicate^25^. Samples collected at diagnosis had been previously analyzed using the same protocol^33^.

Metabolites with inter-batch or intra-batch coefficients of variation (CVs) > 20% for IARC replicates were excluded from the analysis, leading to a total of 149 metabolites. Of these, metabolites with > 20% of missing values and/or measurements outside the measurable range (i.e. below the limit of detection/quantification or above highest calibration standards) were excluded^34^, resulting in a total of 138 metabolites included in the current analysis, including 13 acylcarnitines, 21 amino acids, nine biogenic amines, 80 PCs (11 lysoPCs, 34 diacylPCs, and 35 acyl-alkylPCs), 14 SMs, and a sum of hexoses. Supplementary Table 1 lists measurement information on all metabolites in the kit. Mean (intrabatch) CVs of QC samples from CRC survivors in the previous cross-sectional study were < 20% for all included metabolites. Measurements below the limit of detection or quantification (where applicable) were set to half the batch-specific limit of detection or quantification, respectively^34^. In addition, all measurements above the highest calibration standards were set to the highest standard concentration. Metabolite concentrations (µM) were log-transformed (natural logarithm) to reduce right skewness of distributions.

### Covariates

Highest attained education level was self-reported by participants at diagnosis, as low, medium, or high. At each post-treatment time point, smoking status (never, former or current) was assessed through self-report, weight and height of participants was measured by trained dieticians and used to calculate body mass index (BMI; kg/m^2^), alcohol intake (g/day) was assessed through 7-day dietary records^35^, and the number of comorbidities was measured using the 13-item Self-Administered Comorbidity Questionnaire^36^. Clinical characteristics including tumor site (colon/rectum), cancer stage and treatment were collected from clinical records.

### Statistical analysis

Descriptive analyses were performed for sociodemographic, lifestyle, and clinical variables. Correlation coefficients between physical activity variables and metabolites were visualized in heatmaps. The development of metabolite concentrations during post-treatment measurements was assessed using linear mixed models with batch and sex-adjusted residuals (calculated as described below) as dependent variable, and time since end of treatment included as independent variable (continuous, per 6 months).

To be able to adjust for sex and analytical batch in the main analysis, residuals of each of the ln-transformed metabolite concentrations were computed from linear mixed models with sex as independent variable and random intercepts for individuals nested within analytical batches. The residuals were summed with individual random effects (to retain individual variation in metabolites over time, while excluding batch effects) to be used as dependent variables in further analyses.

Linear mixed regression with random intercepts for individuals was applied to analyze longitudinal associations of self-reported MVPA and LPA (continuously, per 5 h/week) and adherence to the physical activity guideline (yes/no) with circulating concentrations of each of the metabolites (batch and sex-adjusted residuals), between 6 weeks up to 2 years post-treatment. Obtained regression coefficients of the overall longitudinal relationship can be interpreted as a weighted average of a between-subject component (i.e. how differences in physical activity variables *between* participants are related to metabolite concentrations over time: inter-individual associations) and a within-subject component (i.e. how changes in physical activity variables *within* participants over time are related to metabolite concentrations over time: intra-individual associations). We also applied a hybrid modelling method, where the between-subject component was modelled as the mean value of the physical activity variable for each participant across time points, while the within-subject component was modelled as the difference between the physical activity level at each time point and the mean level across time points^37,38^.

Models were adjusted for age (continuous), sex, hospital, time since end of treatment (continuous), smoking status, BMI (continuous), alcohol intake (continuous), and number of comorbidities (0/1/ ≥ 2 comorbidities) at post-treatment measurements. Models were also adjusted for post-treatment levels of MVPA for the analysis of LPA, and vice versa. Potential confounders were identified a priori based on theoretical considerations and literature; other covariates were considered in sensitivity analyses as described below. To adjust for multiple testing, false discovery rate (FDR) adjustment of P-values using the Benjamini–Hochberg method was applied (q-values < 0.05 were considered significant)^39^.

#### Heterogeneity analyses

To explore potential heterogeneity by sex, chemotherapy (yes/no), radiotherapy (yes/no), tumor site, number of comorbidities (≥ 2 versus < 2) and time since end of treatment, interaction product terms (e.g. product of MVPA and sex) were tested with FDR-adjustment.

#### Sensitivity analyses

To assess potential confounding by tumor-related variables, additional analyses were performed including chemotherapy and radiotherapy (yes/no, including neo-adjuvant and/or adjuvant treatment) as covariates. In addition, models were run with additional adjustment for metabolite concentrations at diagnosis, using batch-adjusted residuals that were calculated similarly as for post-treatment data. Based on an a priori defined directed acyclic graph (Supplementary Fig. 1), we hypothesized that adjustment for metabolite concentrations at diagnosis may lead to collider bias, similar to what has been reported previously^40^. Therefore, a difference in results compared to the main analysis could be due to confounding and/or collider bias. Models with additional adjustment for self-reported time spent on LPA and MVPA at diagnosis showed signs of collinearity with increasing standard errors and variance inflation factors of physical activity variables > 2.50^41^, likely due to strong correlations between physical activity variables at diagnosis and at post-treatment measurements (Spearman’s ρ; range: 0.59, 0.80). These models were therefore not reported.

All analyses were conducted using R (version 3.6.2).

### Disclaimer

Where authors are identified as personnel of the International Agency for Research on Cancer / World Health Organization, the authors alone are responsible for the views expressed in this article and they do not necessarily represent the decisions, policy or views of the International Agency for Research on Cancer / World Health Organization.

## Results

### Participant characteristics

Characteristics of included study participants (n = 252) at 6 weeks post-treatment, by adherence to the physical activity guideline, are shown in Table 1. About two-third of the participants were men (68.7%) and the mean age at 6 weeks post-treatment was 66.7 years (SD: 9.2). Mean BMI was 27.8 kg/m^2^ (SD: 4.4). Just over half of participants reported ≥ 2 comorbidities (53.6%) and 25.4% had one comorbidity. Most participants were colon cancer survivors (60.7%), while 39.3% were rectum cancer survivors. The majority of participants received surgery (89.7%), and 38.1% and 27.8% received (neo-)adjuvant chemotherapy and radiotherapy, respectively. Participants who reported adherence to the physical activity guideline at 6 weeks post-treatment (75.8%) were more often men, and reported more often current or former smoking and a higher alcohol intake compared to non-adhering participants; other characteristics were similar.

**Table 1:** Socio-demographic, lifestyle, and clinical characteristics of included participants at 6 weeks post-treatment, by adherence to the physical activity (PA) guideline (≥ 150 min/week of moderate-to-vigorous PA).

	Total number of included participants (n = 252, 100%)^a^	Participants reporting adherence to the PA guideline (n = 191, 75.8%)	Participants reporting non-adherence to the PA guideline (n = 61, 24.2%)
Age, *mean (SD)*	66.7 (9.2)	67.2 (7.6)	65.0 (12.9)
**Sex, *n (%)***			
Men	173 (68.7)	141 (73.8)	32 (52.5)
Women	79 (31.3)	50 (26.2)	29 (47.5)
**Highest attained education level, *n (%)***^**b**^			
Low	64 (25.4)	48 (25.1)	16 (26.2)
Medium	102 (40.5)	78 (40.8)	24 (39.3)
High	86 (34.1)	65 (34.0)	21 (34.4)
Body mass index (kg/m^2^), *mean (SD)*	27.8 (4.4)	27.6 (4.2)	28.5 (5.1)
**Smoking status, *n (%)***			
Current smoker	23 (9.1)	19 (9.9)	4 (6.6)
Former smoker	145 (57.5)	115 (60.2)	30 (49.2)
Never smoker	84 (33.3)	57 (29.8)	27 (44.3)
Alcohol intake in men (g/day), *median (25th, 75th perc)*	10.9 (0.2, 24.4)	12.8 (1.0, 25.9)	6.0 (0.0, 16.0)
Alcohol intake in women (g/day), *median (25th, 75th perc)*	1.5 (0.0, 9.7)	2.9 (0.0, 15.1)	0.0 (0.0, 3.1)
**Number of comorbid conditions, *n (%)***			
0	53 (21.0)	38 (19.9)	15 (24.6)
1	64 (25.4)	52 (27.2)	12 (19.7)
≥ 2	135 (53.6)	101 (52.9)	34 (55.7)
**Tumor site, *n (%)***			
Colon	153 (60.7)	117 (61.2)	36 (59.0)
Rectum	99 (39.3)	74 (38.7)	25 (41.0)
**Colorectal cancer stage, *n (%)***			
I	75 (31.0)	55 (30.0)	20 (33.9)
II	57 (23.6)	45 (24.6)	12 (20.3)
III	110 (45.5)	83 (43.4)	27 (45.8)
Received surgery, *n (%)*	226 (89.7)	171 (89.5)	55 (90.2)
Received chemotherapy, *n (%)*	96 (38.1)	75 (39.3)	21 (34.4)
Neo-adjuvant, *n (%)*	48 (19.0)	33 (17.3)	15 (24.6)
Adjuvant, *n (%)*	71 (28.2)	57 (29.8)	14 (23.0)
Received radiotherapy, *n (%)*^*c*^	70 (27.8)	49 (25.7)	21 (34.4)
Neo-adjuvant, *n (%)*	69 (27.4)	49 (25.7)	20 (32.8)
Adjuvant, *n (%)*	1 (< 0.1)	0 (0.0)	1 (0.2)
**Treatment centre *n (%)***			
Maastricht UMC +	153 (60.7)	114 (59.7)	39 (63.9)
VieCuri Medical Center	68 (27.0)	49 (25.7)	19 (31.1)
Zuyderland Medical Centre	31 (12.3)	28 (14.7)	3 (4.9)

*n* number, *perc* percentile, *SD* standard deviation.

^a^ Of included participants, a total of 10 participants had missing data on blood metabolites and/or physical activity and/or covariates at 6 weeks post-treatment and 1 participant had missing data on blood metabolites and/or physical activity and/or covariates at 6 weeks and 6 months post-treatment, but all of these participants had available data at later time points and were therefore included in the analysis.

^b^ Education level was categorized as low (none/primary education/lower vocational training), medium (lower general secondary education/intermediate vocational education), or high (higher general secondary education/higher vocational education/university).

**Figure 2:**
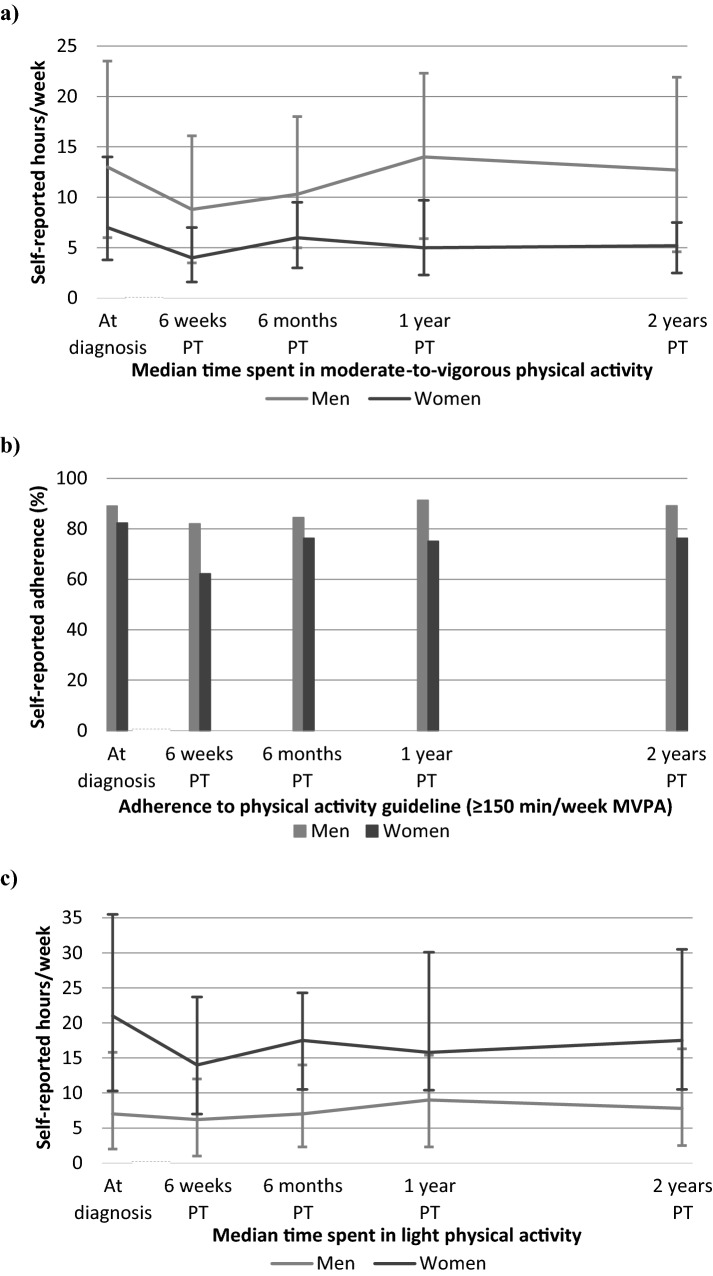
Sex-stratified medians and interquartile ranges of self-reported (**a**) hours/week of moderate-to-vigorous physical activity (MVPA), (**b**) percentage self-reported adherence to physical activity guideline (≥ 150 min/week of MVPA), and (**c**) hours/week of light-intensity physical activity (LPA) at diagnosis and at post-treatment (PT) time points among colorectal cancer survivors included in the current analysis (n = 252).

At each time point, median MVPA was lower among women than men, while the opposite was observed for LPA (Fig. 2). At 6 weeks post-treatment, women reported a median of 4 h/week of MVPA (IQR: 2, 7) and 14 h/week^7,24^ of LPA, while men reported 9 h/week^4,16^ of MVPA and 6 h/week^1,12^ of LPA. Most participants decreased physical activity levels between diagnosis and 6 weeks post-treatment (median change in MVPA in women: − 4, men: − 3 h/week; LPA in women: − 3, men: − 1 h/week), and increased at later post-treatment time points particularly between 6 weeks and 6 months post-treatment (median change in MVPA: + 2 h/week in both sexes; LPA in women: + 2, men: + 0.1 h/week). Both MVPA and LPA showed moderate to strong correlations across repeated measurements (range in Spearman’s ρ for MVPA: 0.58, 0.76; LPA: 0.57, 0.80), while mostly negative and weak correlations were observed between MVPA and LPA at each time point (-0.29, 0.03) (heatmap in Supplementary Fig. 2).

### Metabolome characteristics

Descriptives of included metabolites at each time point are reported in Supplementary Table 2, and heatmaps of correlations between metabolites are depicted in Supplementary Fig. 3. Within classes of metabolites at 6 weeks post-treatment, median Spearman’s ρ was generally higher for lysoPCs (0.50; IQR: 0.29, 0.68), diacylPCs (0.48; 0.36, 0.60), acyl-alkylPCs (0.59; 0.50, 0.68) and SMs (0.60; 0.50, 0.69), compared to acylcarnitines (0.32; 0.14, 0.53), amino acids (0.23; 0.13, 0.35) and biogenic amines (0.14; 0.01, 0.26), and similar patterns were observed at other time points. Correlations between repeated metabolite measurements across post-treatment time points were moderate to strong (range in median Spearman’s ρ: 0.62, 0.70; Supplementary Table 3). During the post-treatment period, 23 metabolites significantly changed over time (FDR q-value < 0.05), with mostly increasing concentrations at later time points (median percentage change in metabolite concentrations per 6 months: 1.3, IQR: 0.4, 1.9; Supplementary Table 4).

### Associations of physical activity with metabolites

**Figure 3:**
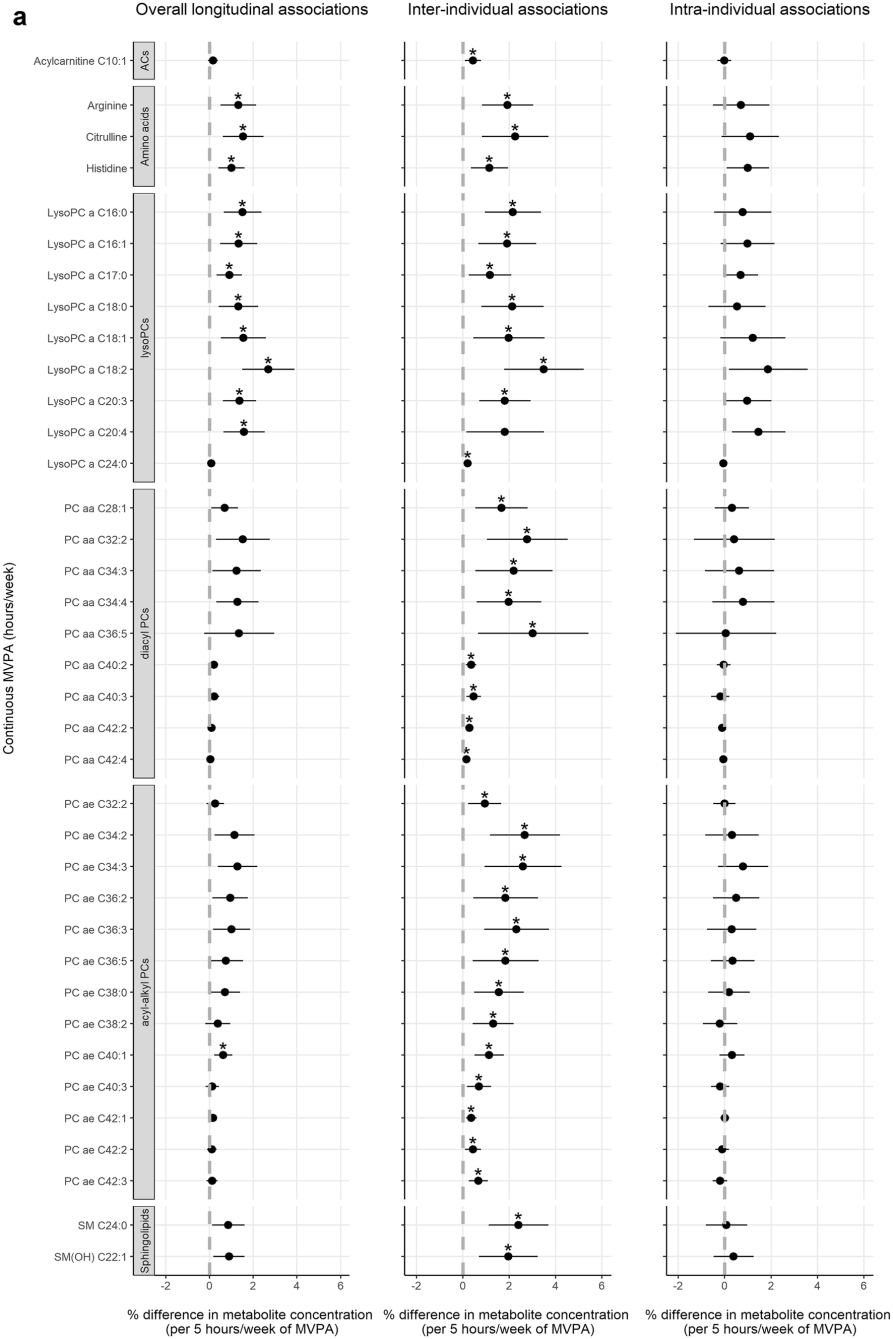

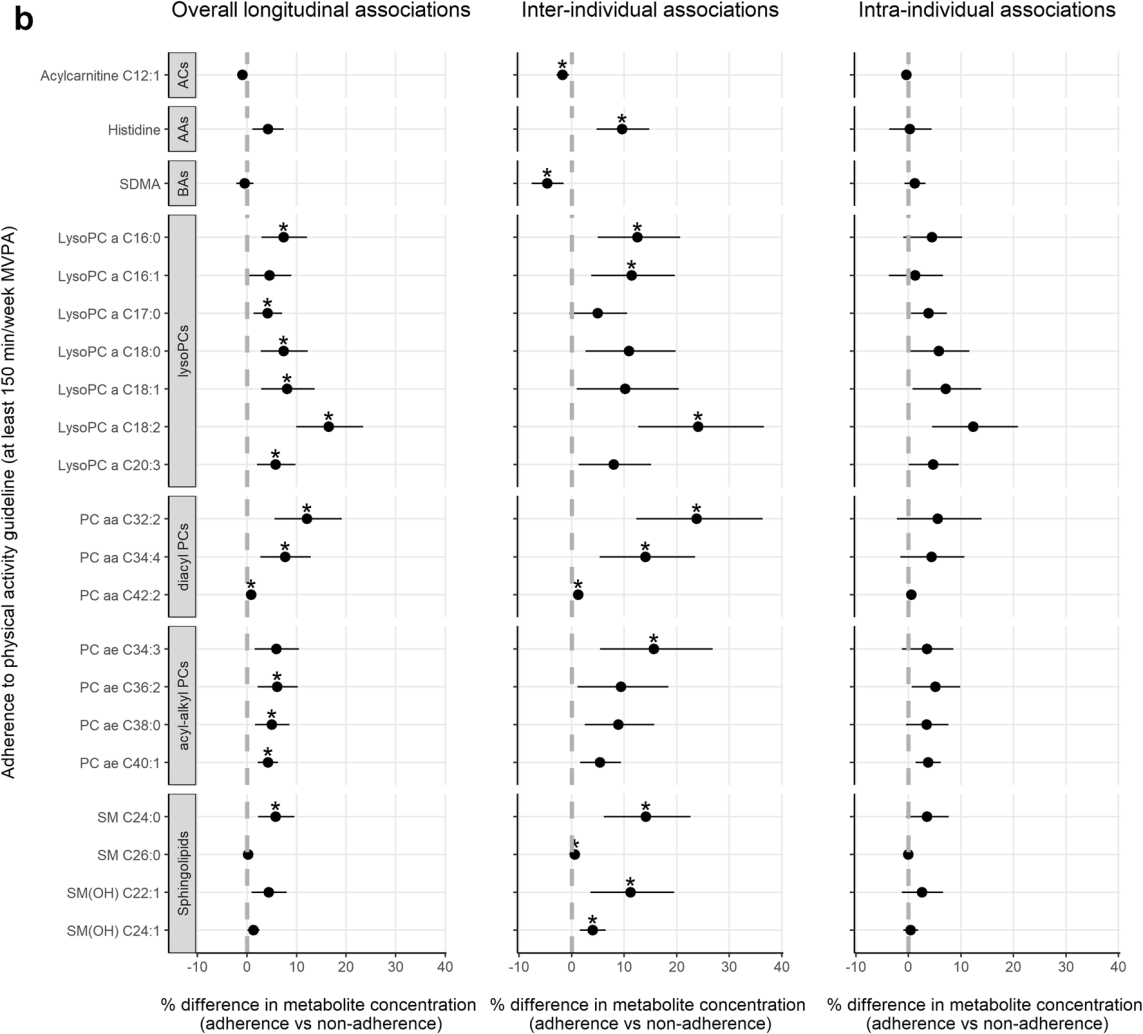
Forest plots showing effect estimates and 95% confidence intervals of metabolites that were statistically significantly (FDR q-value < 0.05) related to self-reported time spent on moderate-to-vigorous physical activity (MVPA; (**a**)) and adherence to the physical activity guideline (≥ 150 min/week of MVPA; (**b**)) among colorectal cancer survivors, between 6 weeks and 2 years post-treatment, including overall, inter-individual and intra-individual longitudinal associations. Asterisk (*) denotes statistical significance after FDR-adjustment (q-values < 0.05 were considered significant). Since light-intensity physical activity (LPA) was not statistically significantly associated with any of the metabolites, these results are not shown. Full results for all physical activity variables and metabolites are included in Supplementary Table 5. *AAs* amino acids, *ACs* acylcarnitines, *BAs* biogenic amines, *MVPA* moderate-to-vigorous physical activity, *PCs* phosphatidylcholines. Analyzed with multivariable linear mixed regression models analyzing associations of the physical activity variables as the main independent variables and as dependent variables the batch-adjusted metabolite residuals (see “Methods” section), with a separate model for each metabolite. Models were adjusted for: sex; age (y; continuous), time since treatment (per 6 months; continuous), centre (Maastricht UMC + ; VieCuri Medical Center; Zuyderland Medical Centre), body mass index (kg/m^2^; continuous), smoking status (current; former; never), self-reported alcohol consumption (grams/day) and number of comorbidities (no comorbidity; 1 comorbidity; ≥ 2 comorbidities), at post-treatment time points. Models were also adjusted for post-treatment levels of MVPA for the analysis of LPA, and vice versa. The percentage difference in metabolite concentrations was calculated by subtracting the exponent of the obtained regression coefficient from 1 and multiplying the outcome with 100 (since metabolite concentrations were ln-transformed).

**Figure 4:**
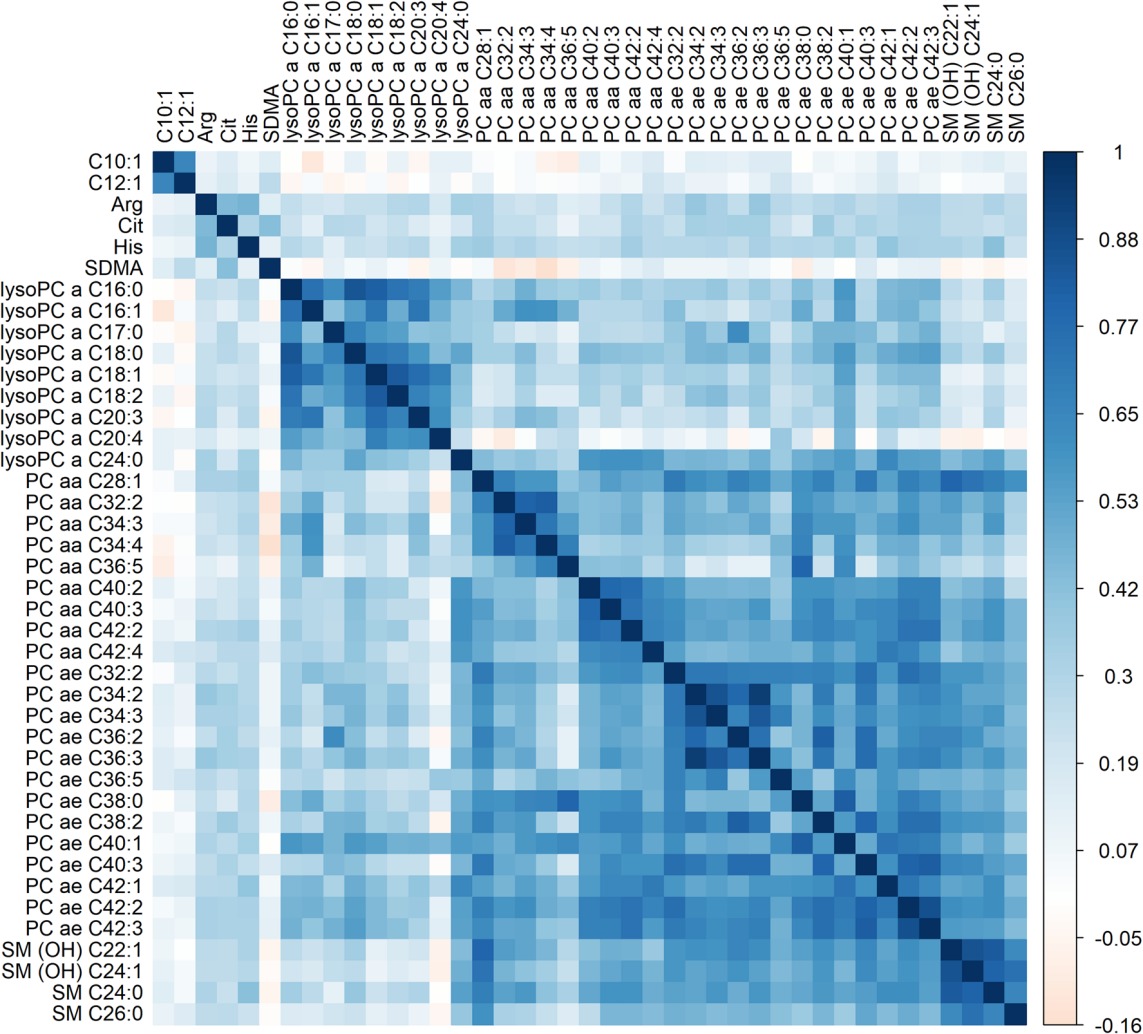
Heatmap of Spearman’s correlation coefficients at 6 weeks post-treatment between plasma concentrations of metabolites that were statistically significantly (FDR q-value < 0.05) related to self-reported time spent on moderate-to-vigorous physical activity (MVPA) and/or adherence to the physical activity guideline (≥ 150 min/week of MVPA) among colorectal cancer survivors. Similar patterns of correlations were observed at other post-treatment time points (results not shown). Heatmap of correlations among all analyzed metabolites at each time point is depicted in Supplementary Fig. 4.

In confounder-adjusted analyses assessing overall longitudinal associations over time, higher levels of MVPA were related to higher concentrations of 12 out of 138 metabolites after FDR-adjustment (Fig. 3a; full results in Supplementary Table 5). In the hybrid model, inter-individual associations were observed with higher levels of MVPA being related to higher plasma concentrations of 36 metabolites, including acylcarnitine C10:1, three amino acids (arginine, histidine and citrulline), eight lysoPCs, nine diacylPCs, 13 acyl-alkylPCs, and two SMs. No statistically significant intra-individual associations were observed and effect estimates were generally smaller than for inter-individual associations. Adherence to the physical activity guideline was associated with higher concentrations of 13 metabolites in the overall longitudinal analysis (Fig. 3b; Supplementary Table 5). In the inter-individual analysis, adherence was associated with higher concentrations of 12 metabolites, including SM C26:0 and SM(OH) C24:1 which had not been identified in the analysis of continuous MVPA. Adherence was also associated with lower concentrations of acylcarnitine C12:1 and symmetric dimethylarginine (SDMA). LPA was not longitudinally associated with any of the metabolites (Supplementary Table 5). Figure 4 shows a heatmap of the correlations at 6 weeks post-treatment across metabolites that were longitudinally related to continuous MVPA and/or adherence to the guideline. Generally, moderate to strong positive correlations were observed within and across classes of PCs and SMs, except for lysoPCs being weakly correlated with SMs and other PCs, and similar patterns were observed at other post-treatment time points.

### Heterogeneity analyses

No statistically significant interactions were observed by sex, chemotherapy, radiotherapy, number of comorbidities (≥ 2 versus < 2) and time since end of treatment (results not shown). One significant interaction was found between tumor site and intra-individual associations of MVPA with PC aa C36:1, where more MVPA was associated with higher concentrations in colon cancer survivors and lower concentrations in rectum cancer survivors.

### Sensitivity analyses

Results of the analysis with additional adjustment for chemotherapy and radiotherapy treatment were similar to those obtained in the main analysis (Supplementary Fig. 4). Adjustment for metabolite concentrations at diagnosis resulted in some attenuation of inter-individual associations for continuous MVPA and adherence to the physical activity guideline (Supplementary Fig. 5).

## Discussion

To our knowledge, this is the first study to analyze longitudinal associations of physical activity with metabolites in CRC survivors. We found that CRC survivors who engaged in more MVPA had higher plasma concentrations of acylcarnitine C10:1, arginine, histidine and citrulline, and eight lysoPCs, nine diacyl PCs, 13 acyl-alkyl PCs, and two SMs. Adherence to the physical activity guideline was additionally related to higher concentrations of two SMs and histidine, and lower concentrations of acylcarnitine C12:1 and SDMA. Contrary to our hypothesis, intra-individual associations for both MVPA variables were weak and statistically non-significant, and self-reported LPA was not longitudinally associated with any of the metabolites.

Our observations of MVPA being positively associated with several mutually correlated PCs and SMs, are in contrast with findings from a population-based study in Germany where self-reported physical activity was not associated with PCs and SMs, measured using a similar targeted metabolomics approach (BIOCRATES p150)^24^. Next to the difference in study population compared to our study (general population *versus* CRC survivors), the differences in study findings may be due to a difference in physical activity assessment, since the German study only included activity during sports^24^, and in our study we used a more comprehensive assessment including also commuting, household, work and other leisure time activities. However, our results are consistent with those of another German study where a higher cardiorespiratory fitness was associated with higher serum concentrations of a cluster of PCs and some SMs, also measured using the BIOCRATES p150^17^. Further, in a US study, Ding et al.^18^ observed that more self-reported MET-hours/week of habitual physical activity was associated with higher plasma concentrations of PCs including lysoPCs, measured using another MS-based platform. Ding et al.^18^ hypothesized that the increase in phospholipids in plasma may be explained by stimulating effects of physical activity on cholesterol efflux through high-density lipoprotein (HDL). A low HDL concentration is one of the features of metabolic syndrome that is associated with worse prognosis after CRC^42^. In addition, PCs can have anti-inflammatory effects^17^ and could therefore mediate the health effects of physical activity after CRC through inflammatory processes^13^.

The amino acids that we identified to be related to MVPA among CRC survivors have previously been associated with physical activity in the general population, although results have been inconsistent. Consistent with our findings, a higher accelerometer-assessed total physical activity duration has been associated with higher plasma concentrations of arginine in Chinese adults^19^, and a higher PAEE has been associated with higher serum concentrations of arginine in Canadian women^23^. Further, positive associations between self-reported physical activity and plasma concentrations of citrulline have been observed in US adults^18^ and accelerometer-assessed MVPA has been associated with higher serum concentrations of histidine in UK adolescents^43^. However, a German study reported that more self-reported physical activity was related to lower serum arginine concentrations in men^24^. In addition, multiple other studies in the general population reported no associations of several physical activity measures with blood concentrations of arginine^17,18,20^, citrulline^17,19,20,24^, and histidine^17–20,23,24^. To our knowledge, no study to date has reported associations of habitual physical activity with SDMA. Experimental studies suggest that arginine intake may protect against CRC (recurrence) by inhibiting crypt cell hyperproliferation, and promoting immune status through increasing the conversion of arginine to nitric oxide (NO)^44^. Citrulline can act as a precursor for arginine and thereby contribute to NO synthesis^45^. SDMA on the other hand has proinflammatory effects and may indirectly inhibit NO production through interfering with arginine transport into cells^46^. Further, anti-inflammatory effects of histidine in intestinal epithelial cells have been reported^47^, and a lower histidine has been associated with poor survival after CRC^48^.

Finally, we observed opposite associations of both MVPA variables with acylcarnitines C10:1 and C12:1. Previous population-based studies reported no significant associations of physical activity with acylcarnitines^17,24^. Acylcarnitines are used as a substrate for mitochondrial oxidation of fatty acids^49^, and are markers for incomplete fatty acid oxidation linked to insulin resistance, type 2 diabetes and cardiovascular health^50^. Experimental studies have found that exercise can induce temporary increases in circulating medium-chain acylcarnitines (including C10:1 and C12:1) as a marker of incomplete fatty acid oxidation^51,52^, but whether habitual physical activity may also have long-term effects on circulating acylcarnitines was yet unknown. Further research will be necessary to determine whether these acylcarnitines may be related to the health of CRC survivors.

We observed inter-individual associations of MVPA with several metabolites, but intra-individual associations were weak and non-significant. This suggests that post-treatment levels but not changes in MVPA are associated with metabolic health in CRC survivors, which may be relevant in light of potential development of interventions. One explanation of our findings is that the intra-individual changes in MVPA and circulating metabolite concentrations over time may have been insufficient, as correlations between repeated measurements were strong and observed changes in physical activity and metabolites were generally limited. Only seven of the metabolites for which inter-individual associations were observed with the MVPA variables were found to significantly change over time. Our analysis was limited to metabolites included in the kit, of which the majority has been previously observed to be stable over time^30,31^. Further experimental studies with larger contrasts in physical activity and including also other metabolites (e.g. using untargeted approaches) are necessary to further investigate whether changes in MVPA may influence metabolic pathways in CRC survivors.

Strengths of our study include the prospective design of our study with repeated measurements of physical activity, metabolomics, and potential confounders. Using a hybrid model, we were able to distinguish between intra- and inter-individual associations over time. Finally, all post-treatment samples were drawn under fasting conditions and stored and analyzed according to standardized protocols, ensuring the quality and comparability of metabolomics data. There are also limitations to consider. Firstly, physical activity levels were self-reported through a questionnaire which is prone to recall errors, and a limited validity of the SQUASH has been observed particularly for LPA^29^. The SQUASH mainly assesses light (household) work as LPA and no other light-intensity activities such as light-walking or standing during leisure time. This may have contributed to the limited intra-individual variation observed over time in LPA, and the lack of longitudinal associations between LPA and metabolites. Further research with accelerometer data will be necessary to confirm our findings. In addition, we were not able to adjust our analysis for physical activity levels at diagnosis, because of collinearity in these models. Therefore, we cannot rule out the possibility that the associations we observed of MVPA with metabolites may reflect effects of lifetime MVPA rather than (only) post-treatment MVPA levels. Further, apart from alcohol consumption and BMI, which are both strongly related to the metabolites included in the BIOCRATES kit^34,53^, we did not adjust our analysis for other dietary(-related) factors and this could have resulted in residual confounding. In addition, collider bias may have occurred in the analysis with adjustment for metabolite concentrations at diagnosis, which could have caused the attenuation observed. Nevertheless, the attenuation was limited and results were still in the same direction, indicating the robustness of our results.

In conclusion, we observed in this longitudinal study that self-reported time spent on MVPA was associated with plasma concentrations of several PCs, SMs, arginine, citrulline, histidine, SDMA and two acylcarnitines in CRC survivors. Our results indicate that differences in (long-term) MVPA levels between CRC survivors—but not intra-individual changes in MVPA after treatment—are associated with post-treatment metabolite concentrations. These results point towards potential underlying biological mechanisms including inflammation and other metabolic pathways, which may underlie the health-enhancing effects of (lifetime) physical activity in CRC survivors. Further longitudinal accelerometer-based studies are necessary to confirm our results, as well as mechanistic studies to investigate whether these metabolites may affect prognosis after CRC.

## Supplementary Information

Supplementary Information 1.

Supplementary Information 2.

## Data Availability

The datasets generated during and/or analysed during the current study are available from the corresponding author on reasonable request.
