# Supplementary material for: Longitudinal associations of physical activity with plasma metabolites among colorectal cancer survivors up to 2 years after treatment
Source: Sci Rep. 2021 Jul 2;11:13738. doi: 10.1038/s41598-021-92279-9 (PMC8253824; doi:10.1038/s41598-021-92279-9)
Supplement: Supplementary file 2 — Supplementary Information 2. [file 41598_2021_92279_MOESM2_ESM.docx]

**Longitudinal associations of physical activity with plasma metabolites among colorectal cancer survivors up to two years after treatment**

Eline H. van Roekel^1*^, Martijn J.L. Bours^1^, Linda van Delden^2^, Stéphanie O. Breukink^3^, Michèl Aquarius^4^, Eric T.P. Keulen^5^, Audrey Gicquiau^6^, Vivian Viallon^7^, Sabina Rinaldi^6^, Paolo Vineis^8,9^, Ilja C.W. Arts^10,11^, Marc J. Gunter^12^, Michael F. Leitzmann^13^, Augustin Scalbert^6^, Matty P. Weijenberg^1^

**Supplementary Figures**

Unmeasured factors influencing metabolite concentrations (e.g. inflammatory factors)

Metabolite concentrations at colorectal cancer diagnosis

Metabolite concentrations at post-treatment time points

Physical activity at colorectal cancer diagnosis

Physical activity at
post-treatment time points

**Supplementary Figure 1**. Directed acyclic graph (DAG) showing potential collider bias in the analysis of associations of physical activity at post-treatment time points with metabolite concentrations at post-treatment time points (analysis presented in the current manuscript), caused by adjustment for metabolite concentrations at colorectal cancer diagnosis. Metabolite concentrations at colorectal cancer diagnosis are hypothesized to be influenced by unmeasured factors influencing metabolite concentrations as well as by physical activity levels at colorectal cancer diagnosis. Thus, metabolite concentrations at colorectal cancer diagnosis is a collider on this path (common effect of two variables). Adjustment for this collider opens the path between unmeasured factors influencing metabolite concentrations at colorectal cancer diagnosis and physical activity at colorectal cancer diagnosis. Because the unmeasured factors influencing metabolite concentrations are also related to metabolite concentrations at post-treatment time points and because physical activity at colorectal cancer diagnosis is also related to physical activity at post-treatment time points, this may lead to collider bias in the analysis of physical activity at post-treatment time points and metabolite concentrations at post-treatment time points. This effect is similar to what has been previously reported by Glymour *et al*. (Am J Epidemiol, 2005. 162(3): p. 267-78). Blocking of the path between metabolite concentrations at colorectal cancer diagnosis and physical activity at post-treatment time points through adjustment for physical activity at colorectal cancer diagnosis was not possible due to potential collinearity (see Methods section of manuscript). Physical activity at colorectal cancer diagnosis may influence (some of) the unmeasured factors influencing metabolite concentrations, but this arrow was not depicted for clarity since adjustment for both physical activity at diagnosis and these unmeasured factors was not possible and therefore collider bias may still occur.


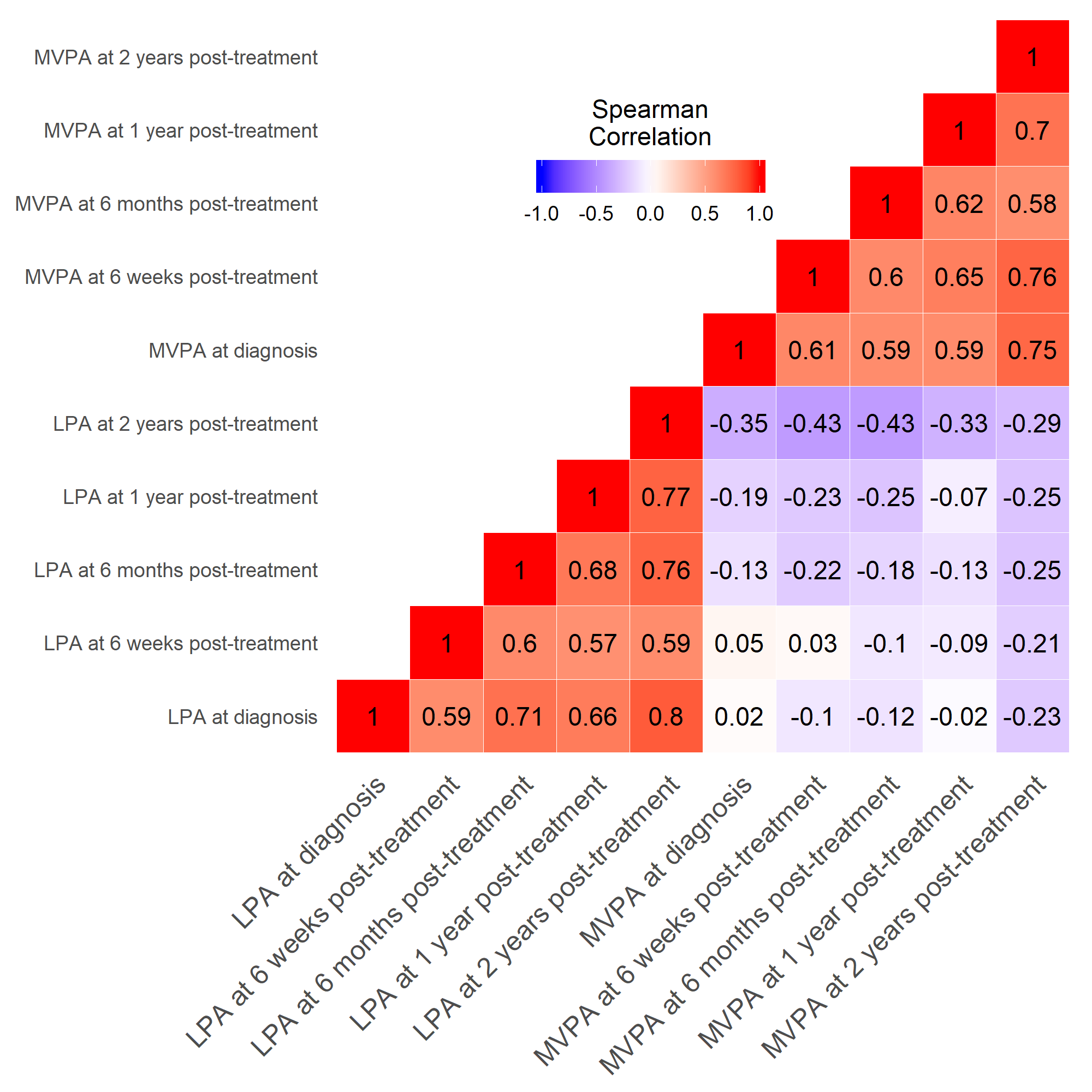


**Supplementary Figure 2**. Heatmap of Spearman’s correlation coefficients between self-reported time spent on light-intensity physical activity (LPA) and moderate-to-vigorous physical activity (MVPA) in hours/week among included colorectal cancer survivors, at colorectal cancer diagnosis and at post-treatment time points between 6 weeks and 2 years post-treatment.


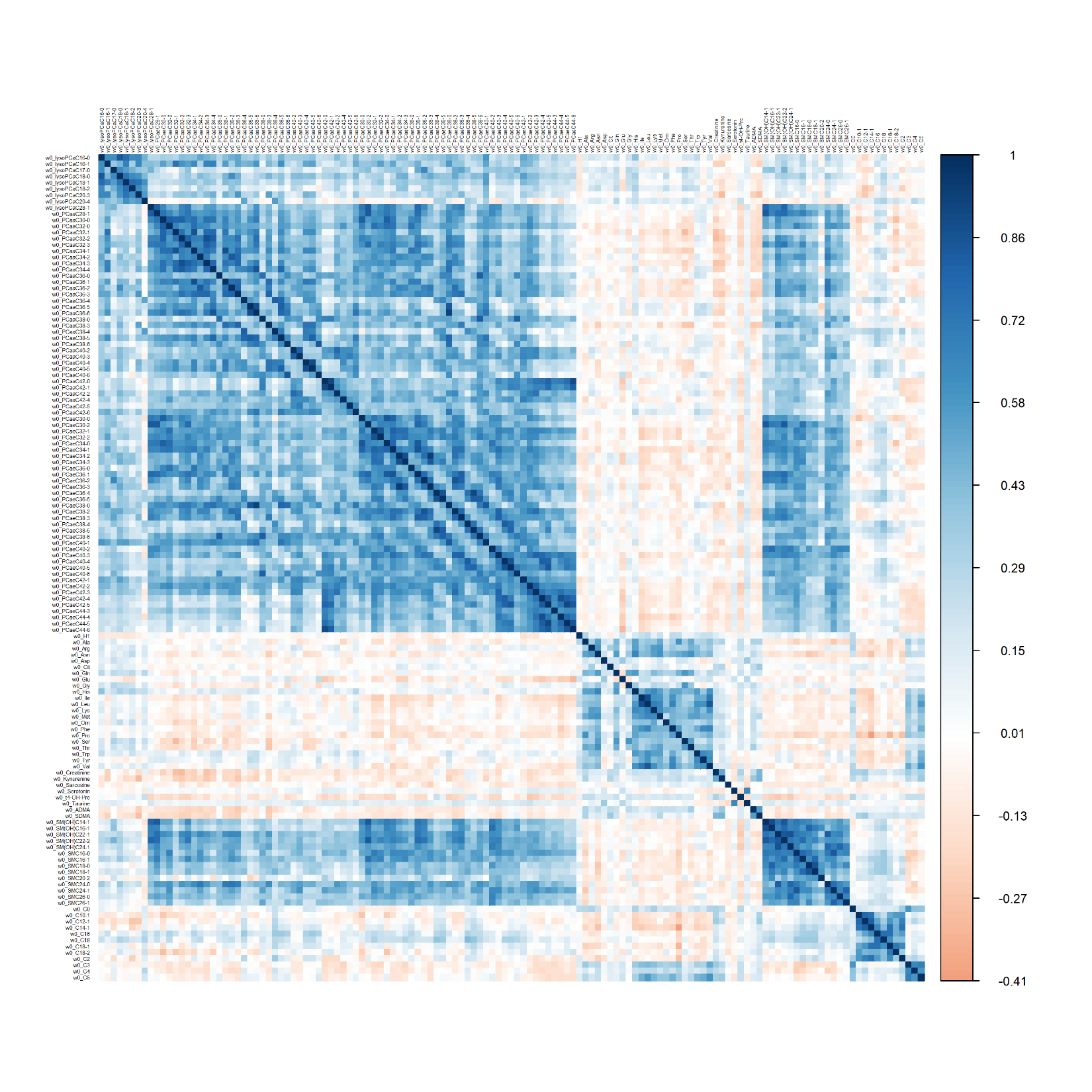


**Supplementary Figure 3a**. Heatmap of Spearman’s correlation coefficients between metabolite concentrations measured at colorectal cancer diagnosis. Heat maps for post-treatment time points are shown on the following pages.

**
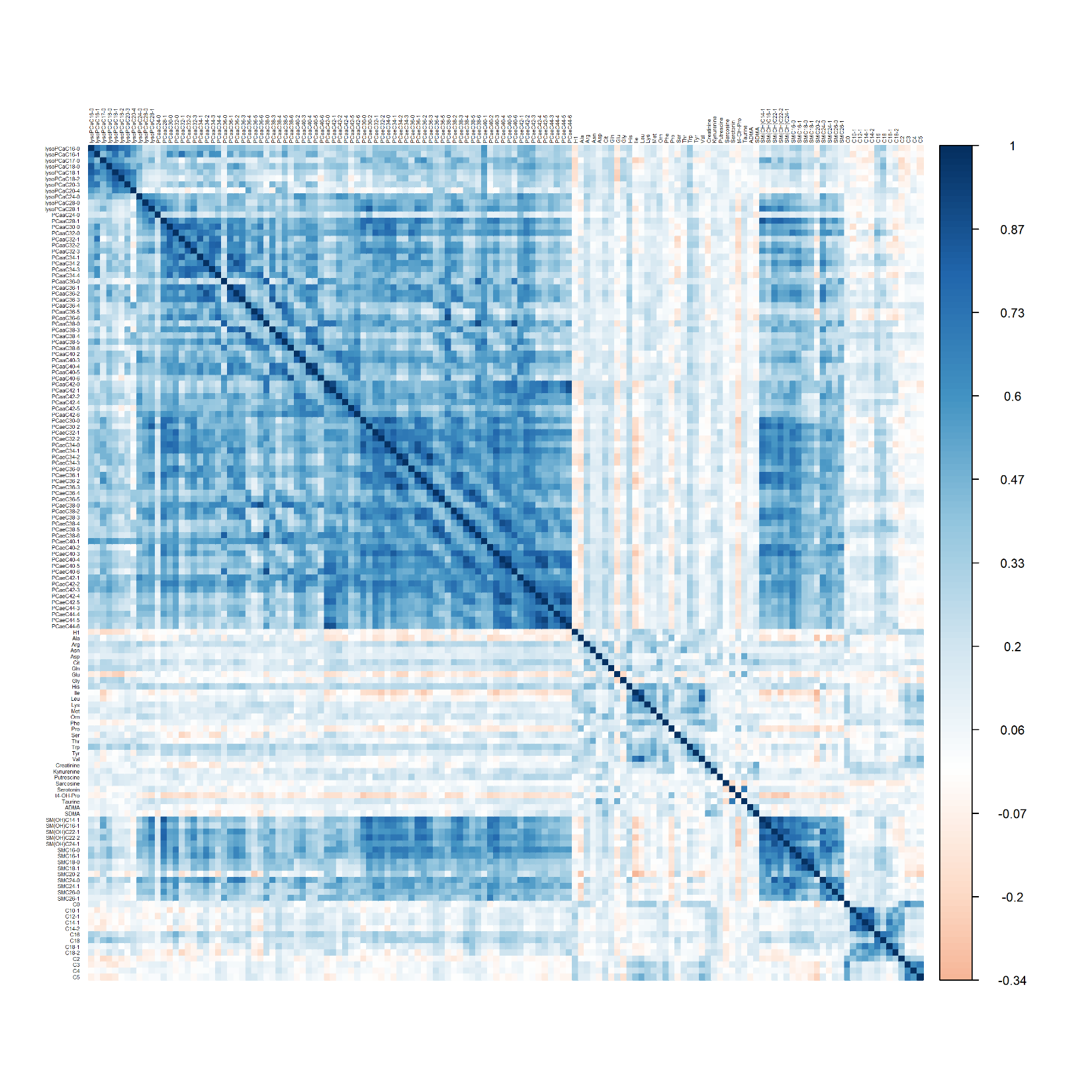
**

**Supplementary Figure 3b**. Heatmap of Spearman’s correlation coefficients between metabolite concentrations measured at 6 weeks post-treatment.


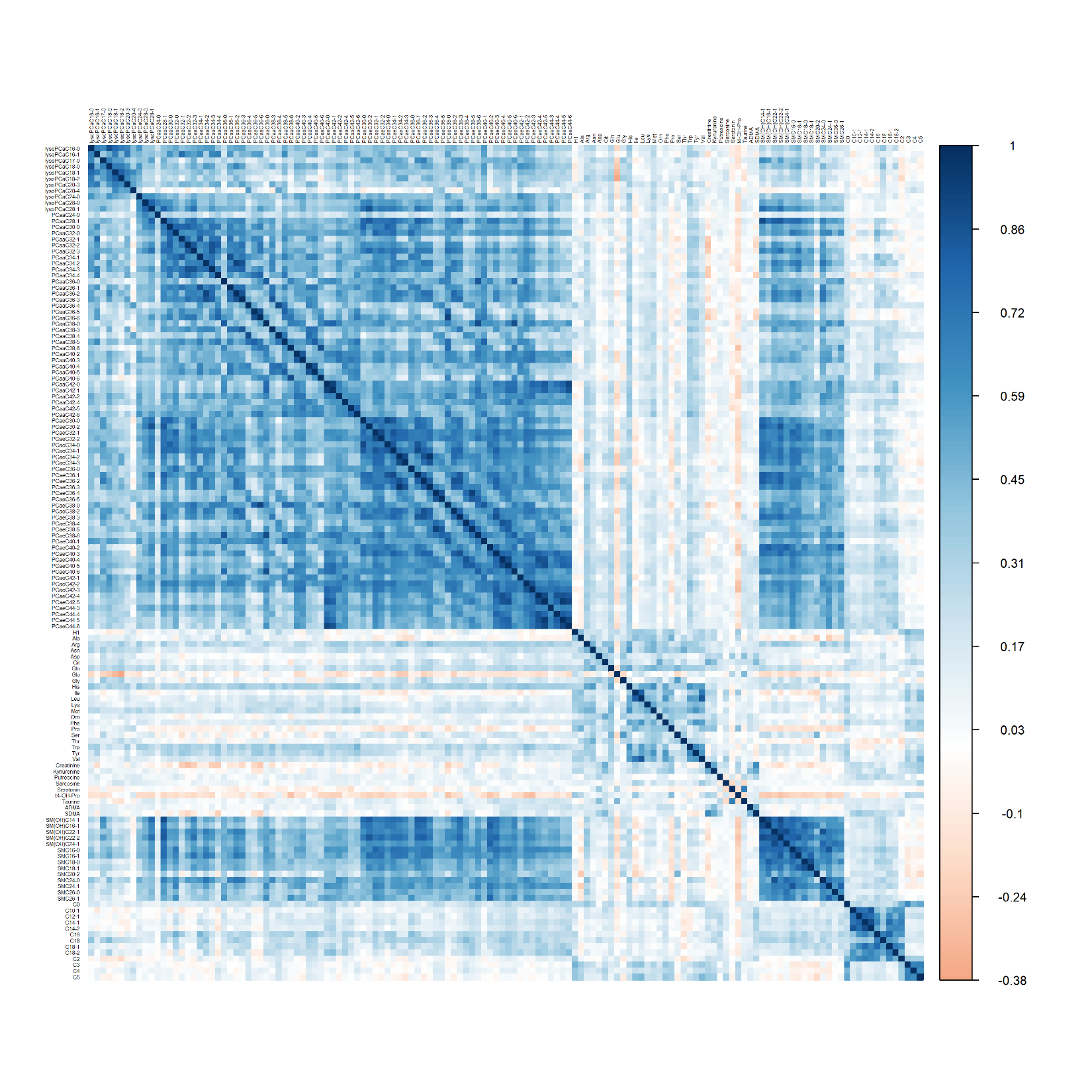


**Supplementary Figure 3c**. Heatmap of Spearman’s correlation coefficients between metabolite concentrations measured at 6 months post-treatment.

**
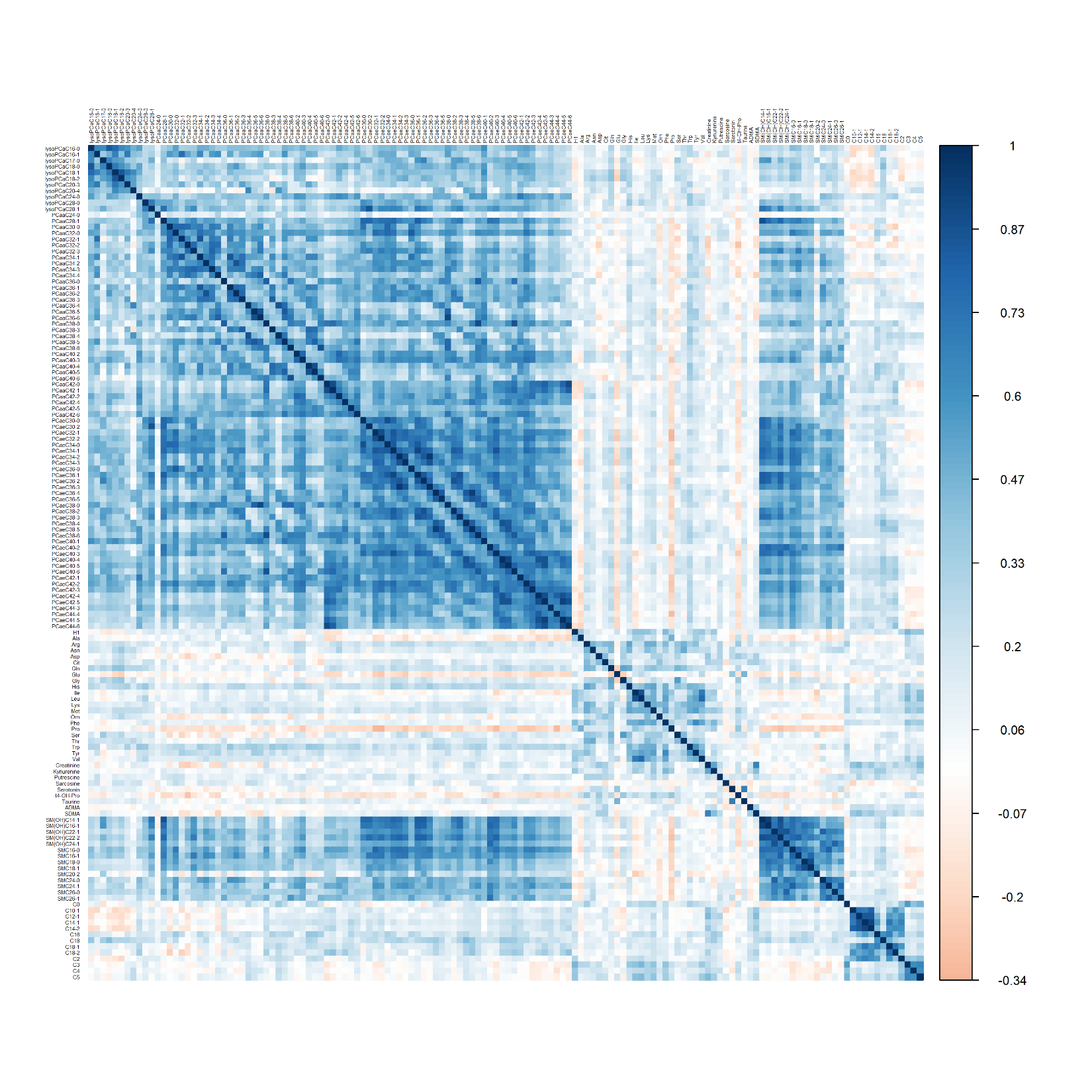
**

**Supplementary Figure 3d**. Heatmap of Spearman’s correlation coefficients between metabolite concentrations measured at 1 year post-treatment.

**
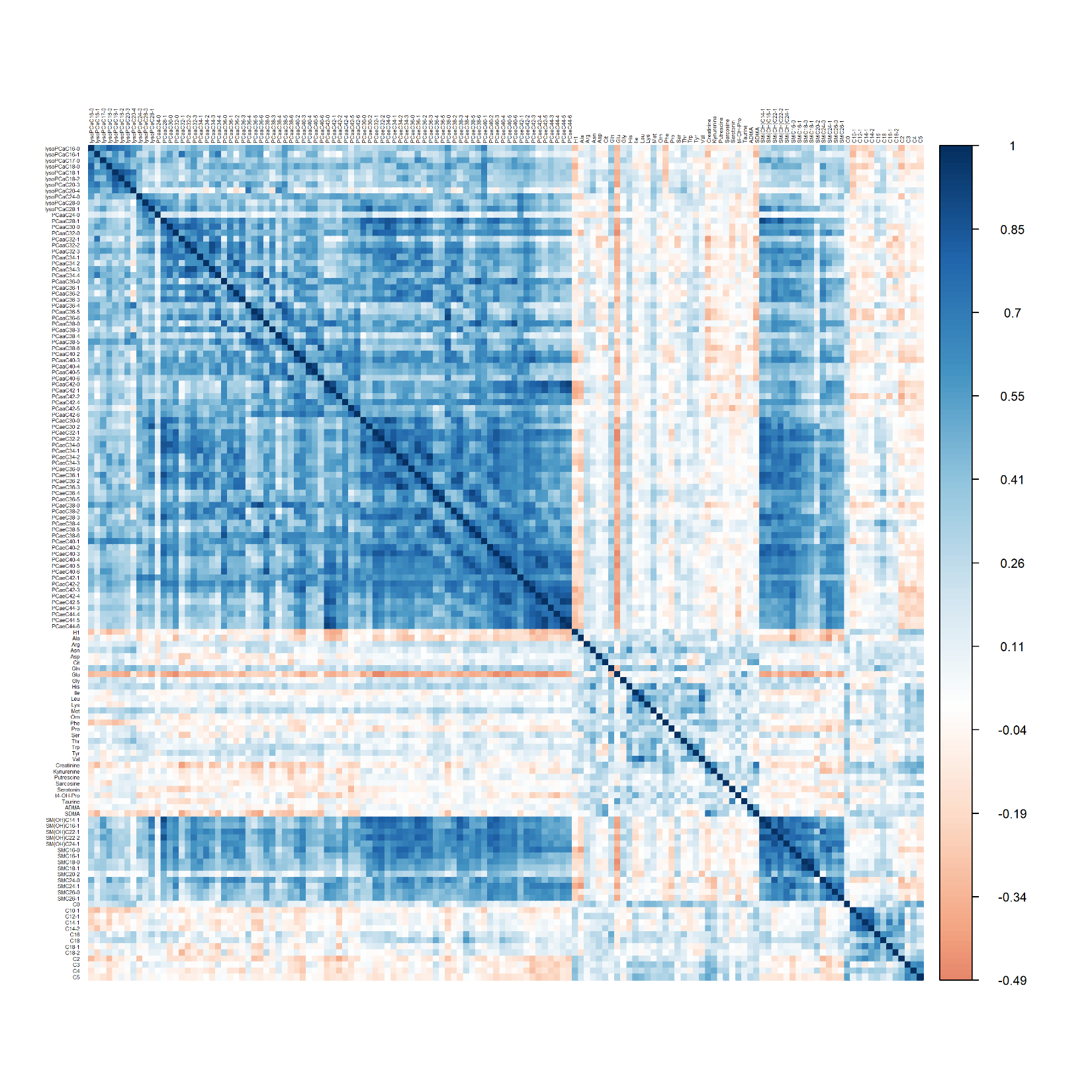
**

**Supplementary Figure 3e**. Heatmap of Spearman’s correlation coefficients between metabolite concentrations measured at 2 years post-treatment.


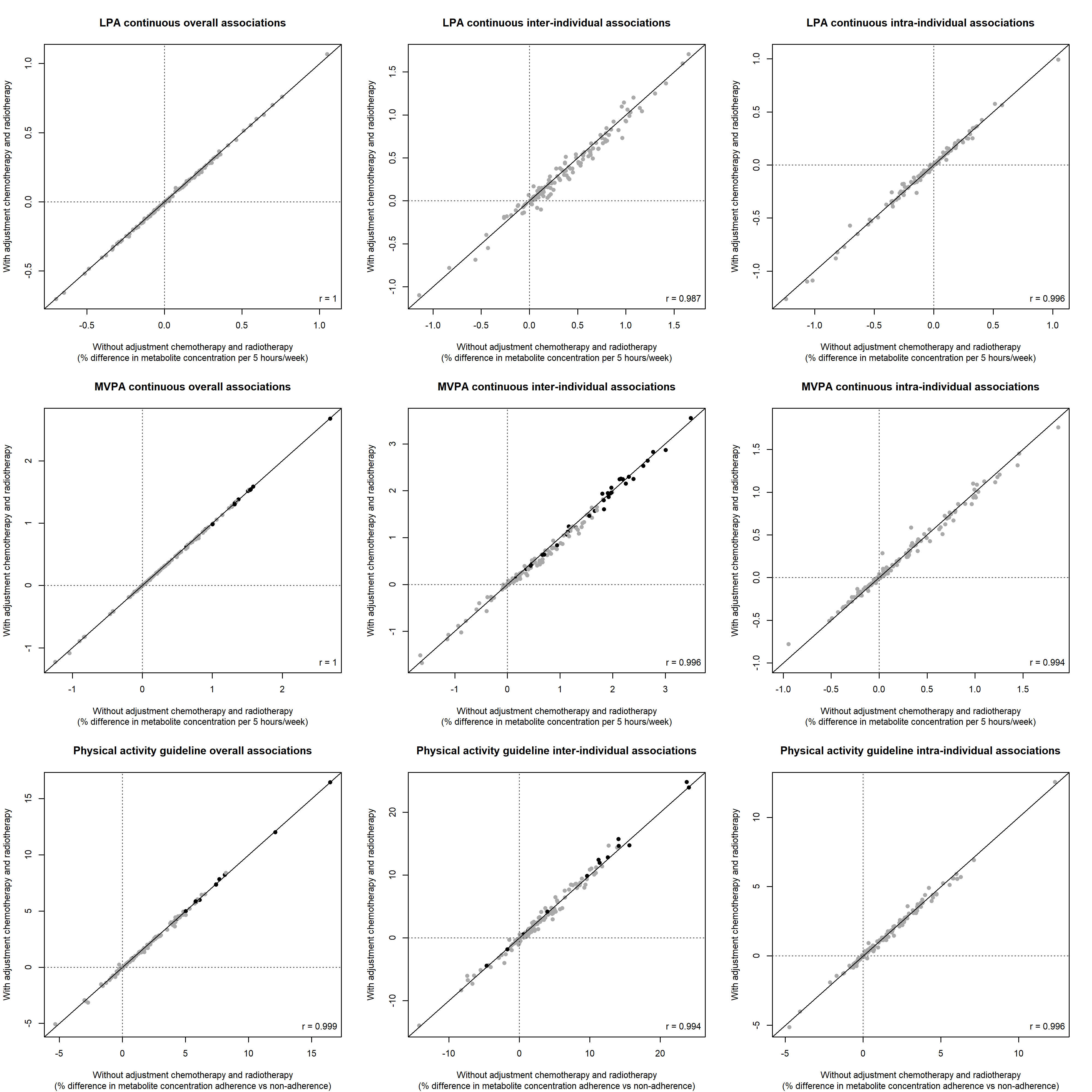


**Supplementary Figure 4**. Comparison figures showing effect estimates derived of linear mixed models assessing longitudinal associations (including overall, intra- and inter-individual associations) of self-reported time spent on light-intensity physical activity (LPA) and moderate-to-vigorous physical activity (MVPA) and adherence to the physical activity guideline (≥150 min/week of MVPA) with metabolite concentrations, among included colorectal cancer survivors between 6 weeks until 2 years post-treatment, for the main analysis (x-axis) and for models with additional adjustment for chemotherapy and radiotherapy treatment (y-axis). Associations that were statistically significant in the main analysis are indicated by black dots, while non-significant associations are presented in grey. The results were analyzed with multivariable linear mixed regression models analyzing associations of the physical activity variables as the main independent variables and as dependent variables the batch-adjusted metabolite residuals (see Methods), with a separate model for each metabolite. Models were adjusted for: sex; age (y; continuous), time since treatment (per 6 months; continuous), centre (Maastricht UMC+; VieCuri Medical Center; Zuyderland Medical Centre), body mass index (kg/m^2^; continuous), smoking status (current; former; never), self-reported alcohol consumption (grams/day) and number of comorbidities (no comorbidity; 1 comorbidity; ≥2 comorbidities), at post-treatment time points. Further, LPA models were adjusted for MVPA (hours/week; continuous) at post-treatment time points and vice versa, and models with the physical activity guideline were adjusted for LPA (hours/week; continuous) at post-treatment time points. The models with additional adjustment included also chemotherapy and radiotherapy treatment (both, yes/no) as covariates. The percentage difference in metabolite concentrations was calculated by subtracting the exponent of the obtained regression coefficient from 1 and multiplying the outcome with 100 (since metabolite concentrations were ln-transformed).


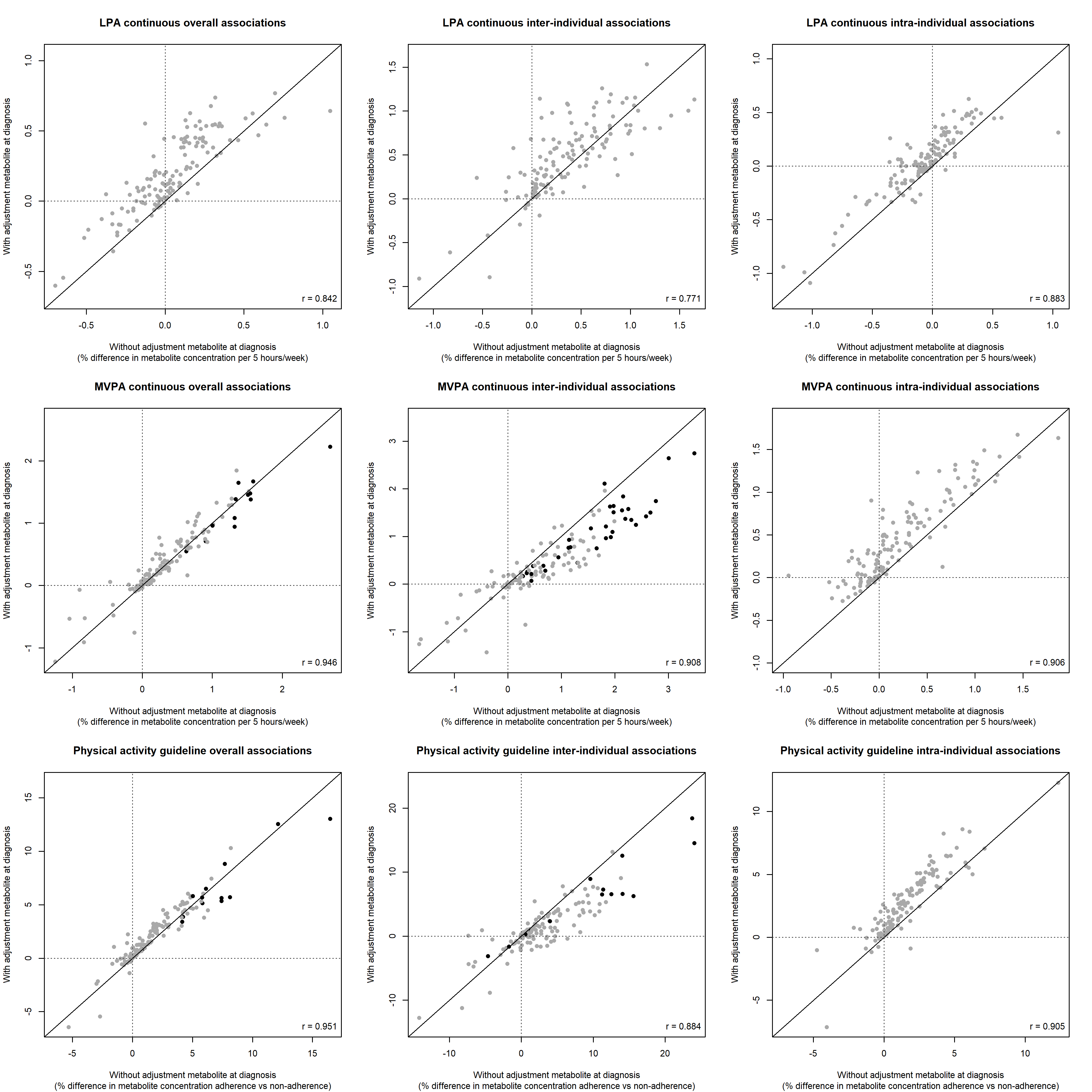


**Supplementary Figure 5**. Comparison figures showing effect estimates derived of linear mixed models assessing longitudinal associations (including overall, intra- and inter-individual associations) of self-reported time spent on light-intensity physical activity (LPA) and moderate-to-vigorous physical activity (MVPA) and adherence to the physical activity guideline (≥150 min/week of MVPA) with metabolite concentrations, among included colorectal cancer survivors between 6 weeks until 2 years post-treatment, for the main analysis (x-axis) and for models with additional adjustment for metabolite concentration at diagnosis (y-axis). Associations that were statistically significant in the main analysis are indicated by black dots, while non-significant associations are presented in grey. The results were analyzed with multivariable linear mixed regression models analyzing associations of the physical activity variables as the main independent variables and as dependent variables the batch-adjusted metabolite residuals (see Methods), with a separate model for each metabolite. Models were adjusted for: sex; age (y; continuous), time since treatment (per 6 months; continuous), centre (Maastricht UMC+; VieCuri Medical Center; Zuyderland Medical Centre), body mass index (kg/m^2^; continuous), smoking status (current; former; never), self-reported alcohol consumption (grams/day) and number of comorbidities (no comorbidity; 1 comorbidity; ≥2 comorbidities), at post-treatment time points. Further, LPA models were adjusted for MVPA (hours/week; continuous) at post-treatment time points and vice versa, and models with the physical activity guideline were adjusted for LPA (hours/week; continuous) at post-treatment time points. The models with additional adjustment included also batch-adjusted metabolite residuals at diagnosis as covariate (see Methods). The percentage difference in metabolite concentrations was calculated by subtracting the exponent of the obtained regression coefficient from 1 and multiplying the outcome with 100 (since metabolite concentrations were ln-transformed).
